# Cardio-oncology in Austria: cardiotoxicity and surveillance of anti-cancer therapies: Position paper of the Heart Failure Working Group of the Austrian Society of Cardiology

**DOI:** 10.1007/s00508-022-02031-0

**Published:** 2022-05-04

**Authors:** Jutta Bergler-Klein, Peter P. Rainer, Markus Wallner, Marc-Michael Zaruba, Jakob Dörler, Armin Böhmer, Tamara Buchacher, Maria Frey, Christopher Adlbrecht, Rupert Bartsch, Mariann Gyöngyösi, Ursula-Maria Fürst

**Affiliations:** 1 Department of Cardiology, University Clinic of Internal Medicine II, Medical University of Vienna, Waehringer Guertel 18–20, 1090 Vienna, Austria; 2 Division of Cardiology, Medical University of Graz, Graz, Austria; 3 BioTechMed Graz, Graz, Austria; 4 Cardiovascular Research Center, Lewis Katz School of Medicine, Temple University, Philadelphia, PA USA; 5 Department of Internal Medicine III, Cardiology and Angiology, Medical University of Innsbruck, Innsbruck, Austria; 6 Department of Internal Medicine and Cardiology, Klinikum Klagenfurt, Klagenfurt, Austria; 7 Department of Internal Medicine 1, Krems University Clinic, Krems, Austria; 8 Imed19-privat, Private Clinical Research Center, Vienna, Austria; 9 Department of Medicine 1, Division of Oncology, Medical University of Vienna, Vienna, Austria; 10 Department of Internal Medicine, Hospital of the Brothers of St. John of God (Krankenhaus Barmherzige Brüder) Salzburg, Salzburg, Austria

**Keywords:** Heart failure, Chemotherapy, Immune checkpoint inhibitors, Radiation therapy, Targeted therapy

## Abstract

Survival in cancer is continuously improving due to evolving oncological treatment. Therefore, cardiovascular short-term and long-term side effects gain crucial importance for overall outcome. Cardiotoxicity not only presents as heart failure, but also as treatment-resistant hypertension, acute coronary ischemia with plaque rupture or vasospasm, thromboembolism, arrhythmia, pulmonary hypertension, diastolic dysfunction, acute myocarditis and others. Recent recommendations have proposed baseline cardiac risk assessment and surveillance strategies. Major challenges are the availability of monitoring and imaging resources, including echocardiography with speckle tracking longitudinal strain (GLS), serum biomarkers such as natriuretic peptides (NT-proBNP) and highly sensitive cardiac troponins. This Austrian consensus encompasses cardiotoxicity occurrence in frequent antiproliferative cancer drugs, radiotherapy, immune checkpoint inhibitors and cardiac follow-up considerations in cancer survivors in the context of the Austrian healthcare setting. It is important to optimize cardiovascular risk factors and pre-existing cardiac diseases without delaying oncological treatment. If left ventricular ejection fraction (LVEF) deteriorates during cancer treatment (from >10% to <50%), or myocardial strain decreases (>15% change in GLS), early initiation of cardioprotective therapies (angiotensin-converting enzyme inhibitors, angiotensin or beta receptor blockers) is recommended, and LVEF should be reassessed before discontinuation. Lower LVEF cut-offs were recently shown to be feasible in breast cancer patients to enable optimal anticancer treatment. Interdisciplinary cardio-oncology cooperation is pivotal for optimal management of cancer patients.

## Introduction

Survival in cancer is continuously improving with evolving oncological therapies. Cardiovascular short-term and long-term side effects therefore gain crucial importance for overall outcome [1]. Recent recommendations have proposed surveillance strategies for the timely detection of cardiotoxicity in patients undergoing chemotherapy or other oncological treatment [2]. Controversy remains about the optimal methods and when to initiate cardiac medication in order to continue life-saving cancer therapies [3]. Major challenges are the availability of monitoring and imaging resources [4]. Furthermore, cancer and heart diseases interact with each other [5]. Interdisciplinary cardio-oncology cooperation is needed to improve the management and long-term outcome of cancer patients and prevent cardiovascular complications of anticancer therapies.

The present paper reviews the current literature for detection and treatment of cardiotoxicity in patients undergoing cancer treatment in the context of the Austrian healthcare setting.

## Definition of cardiotoxicity

Cardiotoxicity due to anti-cancer therapy is not only defined as heart failure (HF), but frequently presents as arrhythmia, e.g. atrial fibrillation (AF), atrioventricular (AV) block or ventricular tachycardia (VT), QT-prolongation, arterial hypertension, plaque rupture with acute coronary syndromes, coronary vasospasm, takotsubo syndrome, thromboembolism, pulmonary hypertension, diastolic dysfunction, HFpEF and myocarditis.

Although different cut-offs have been suggested, in the current ESC position statements [2, 6] cancer therapy-related cardiac dysfunction (CTRCD) is defined as any reduction of LVEF < 50% or > 10% absolute reduction from baseline falling below LVEF 50% (EACVI 53%) considered as the lower limit of normal. In addition, an impairment of > 15% from baseline global longitudinal strain in speckle tracking echocardiography is suggested for early detection of myocardial functional deterioration [6].

The onset of cardiotoxicity has been described as acute, during e.g. chemotherapy, early short-term within 1 year, and long-term after 1 year. Traditionally, cardiotoxicity was characterized as irreversible (type I), e.g. in anthracyclines, due to myocardial cell apoptosis and necrosis, or reversible (type II), e.g. in trastuzumab due to additive stunning of myocardial function; however, these categories are debated, as overlap exists. Cancer therapy related to myocardial injury and dysfunction is a continuous process and is also reflected by increased biomarkers [7, 8].

## Specific potentially cardiotoxic anti-cancer agents

### Anthracyclines

Anthracyclines are cytotoxic drugs with a 4-ring structure that interfere with the DNA and RNA synthesis, leading to cell death of rapidly proliferating cells. Anthracyclines such as doxorubicin, daunorubicin, epirubicin and idarubicin are effective in treating, e.g. leukemia, lymphomas, or solid cancers of the lungs, breast, stomach, uterus, ovaries and bladder [9]. The two main dose-limiting side effects include myelosuppression and cardiotoxicity. Introduction of cytokine treatment with the granulocyte colony-stimulating factor (G-CSF) has facilitated the management of myelosuppression. Multiple mechanisms of cardiotoxicity have been identified including increased oxidative stress with reactive oxygen species (ROS) formation, mitochondrial iron accumulation, and inhibition of topoisomerase II leading to myocardial cell apoptosis [10–12]. In experimental models, transcriptional activation of collagen synthesis and thus fibrogenesis was shown [13]. Typically, myocardial wall thinning, and interstitial fibrosis may develop after anthracycline treatment leading to remodeling and dilated cardiomyopathy, especially in younger patients or children [14].

**Fig. 1:**
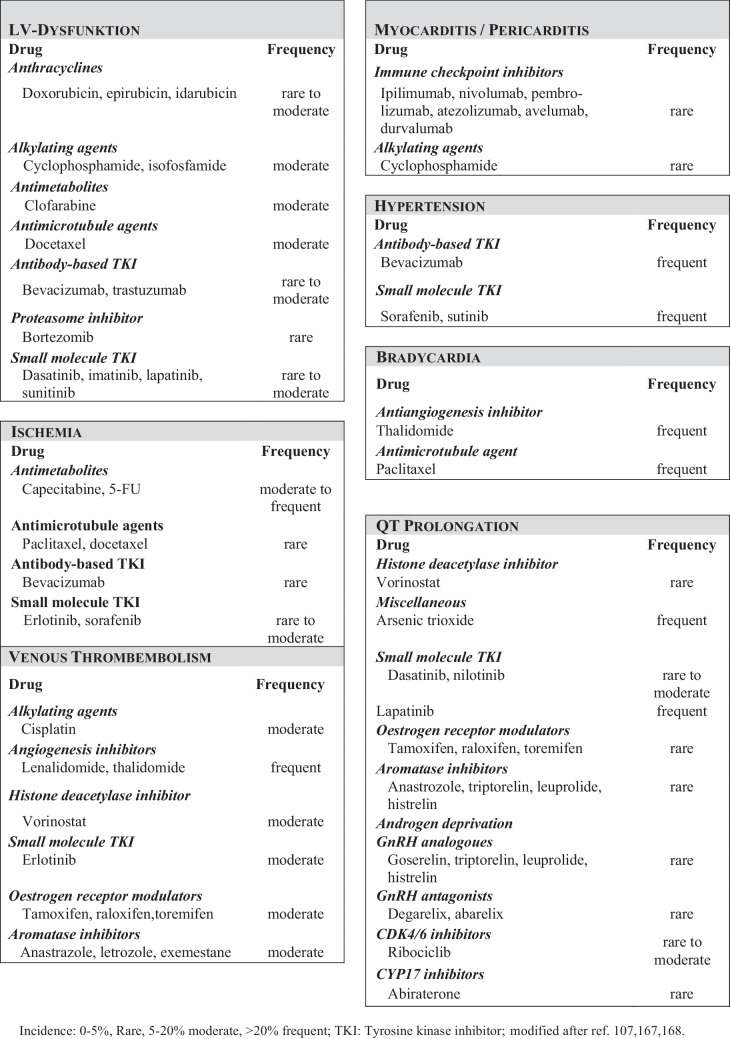
Cardiovascular side effects of anti-neoplastic therapies, overview. Frequency depending on baseline cardiovascular risk and dose of drug (modified after [2, 16, 18, 19]). This is an open access article under the CC BY license (http://creativecommons.org/licenses/by/4.0/). This Table is not included under the Creative Commons CC BY license of this publication

Anthracyclines can cause acute (within 24 h after administration), subchronic (1–3 days), early chronic (< 1 year) and late onset chronic (> 1 year after treatment) cardiac toxicity. The incidence of LV dysfunction and decline in LVEF is dependent on the total cumulative doses and varies, i.e. for doxorubicin from 3–5% at a dose of 400 mg/m^2^ to 18–48% incidence at a dose of 700 mg/m^2^ ([2, 15]; table 1 and Fig. 1). A significant exponential risk increases even at a total anthracycline dose ≥ 250 mg/m^2^ of doxorubicin equivalent.

**Table 1:** Overview of cardiovascular toxicity of anti-neoplastic therapies and what to look for (modified according to [16, 17]). This is an open access article under the CC BY license (http://creativecommons.org/licenses/by/4.0/). This Table is not included under the Creative Commons CC BY license of this publication

Class	Drug	Target	Common cardiovascular toxic effects
*Traditional cancer treatments*			
Alkylating agents	Cyclophosphamide	Cross-link DNA	Congestive heart failure, myocarditis, pericarditis
Antimetabolites	Fluorouracil, capecitabine	Thymidylate synthase	Myocardial ischemia, coronary spasm, arrhythmia
Anthracyclines	Doxorubicin, daunorubicin, idarubicin, epirubicin, mitoxantrone	Type II topoisomerase, DNA and RNA synthesis	CMP, arrhythmia, acute myocarditis or pericarditis
Antimicrotubule agents	Paclitaxel	Microtubule	Arrhythmia (including bradycardia, heart block, PVC, and VT), thrombosis
	Vinca alkaloids	Microtubule	Myocardial ischemia, coronary spasm
Platinum	Cisplatin, carboplatin, oxaliplatin	Cross-link DNA	Hypertension, myocardial ischemia
Radiation	Na	Na	Myocardial ischemia, pericarditis, myocarditis, valvular heart disease, arrhythmia
*Targeted cancer treatments*			
Anaplastic lymphoma kinase inhibitors	Crizotinib, ceritinib	Anaplastic lymphoma kinase	Bradycardia, prolongation of QTc
Bruton’s tyrosine kinase inhibitors	Ibrutinib	Bruton’s tyrosine kinase	Atrial fibrillation, other arrhythmias
HER2 Inhibitors	Trastuzumab, pertuzumab, trastuzumab emtansine, lapatinib	HER2	Decline in LVEF, congestive heart failure
Immunomodulatory drugs	Thalidomide, lenalidomide, pomalidomide	Lymphoid transcription factors IKZF1 and IZKF3	Venous or arterial thromboembolic events
Immune checkpoint inhibitors	Ipilimumab	CTL‑4	Myocarditis, pericarditis, pericardial effusion, conduction disease (AV-block II°/AV-block III°), prolongation of QTc, left ventricular impairment without myocarditis, vasculitis (coronary and large vessel), myocardial infarction, ventricular arrhythmias, takotsubo syndrome
	Nivolumab, pembrolizumab, cemiplimab	PD‑1	
	Atezolizumab, avelumab, durvalumab	PD-L1	
MEK inhibitors	Trametinib	MEK1, MEK2	CMP
Multitargeted tyrosine kinase inhibitors	Dasatinib	ABL, ABL mutants (except T315I), and other kinases; SRC, KIT, PDGFR, EGFR, BRAF, DDR1, DDR2, ephrin receptors	Pulmonary hypertension, vascular events, prolongation of QTc
	Nilotinib	ABL, ABL mutants (except T315I), and other kinases; ABL2, KIT, DDR1, NQO2	Coronary, cerebral, and peripheral vascular events, hyperglycemia, prolongation of QTc
	Ponatinib	ABL, ABL mutants (including T315I), and other kinases; FGFR, VEGFR, PDGFR, ephrin receptors, SRC, KIT, RET, TEK (also called TIE2), FLT3	Coronary, cerebral, and peripheral vascular events
PI3K–AKT–mTOR inhibitors	Everolimus, temsirolimus	PI3K–AKT–mTOR signaling pathway	Cardiometabolic toxic effects, including hypercholesterolemia, hypertriglyceridemia, hyperglycaemia
Proteasome inhibitors	Bortezomib, carfilzomib	Ubiquitin-proteasome system	CMP, hypertension, venous or arterial thromboembolic events, arrhythmia
*VEGF signaling pathway inhibitors*		VEGF signaling pathway	Hypertension, venous or arterial thromboembolic events, proteinuria, cardiomyopathy
VEGFA monoclonal antibody	Bevacizumab		
VEGF trap	Aflibercept		
VEGFR2 monoclonal antibody	Ramucirumab		
Tyrosine kinase inhibitor with anti-VEGF activity	Sunitinib, sorafenib, pazopanib, axitinib, vandetanib, regorafenib, cabozantinib, lenvatinib	VEGF receptors (mainly VEGFR2) and other kinases; PDGFR	

*CMP* cardiomyopathy, *PVC* premature ventricular complexes, *VT* ventricular tachycardia, *na* not applicable, *HER2* human epidermal growth factor receptor 2, *LVEF* left ventricular ejection fraction, *IKZF* IKAROS family zinc finger, *CTLA4* cytotoxic T-lymphocyte–associated protein 4, *AV-block* atrioventricular block, *PD1* programmed cell death protein, *PD-L1* programmed cell death protein ligand 1, *MEK* mitogen-activated protein kinase, *ABL* Abelson murine leukemia viral oncogene homolog, *PDGFR* platelet-derived growth factor receptor, *EGFR* epidermal growth factor receptor, *DDR1 and DDR2* discoidin domain receptor family, members 1 and 2, *NQO2* NAD(P)H quinone dehydrogenase 2, *FGFR* fibroblast growth factor receptor, *VEGFR* vascular endothelial growth factor receptor, *FLT3* fms-related tyrosine kinase 3, *PI3K* phosphatidylinositol 3‑kinase, *AKT* protein kinase B, *mTOR* mammalian target of rapamycin

Risk factors for anthracycline-related cardiotoxicity include age > 65 years or < 18 years, female sex, baseline LV dysfunction (asymptomatic LVEF < 50%, or high natriuretic peptide), prior cardiovascular disease (e.g. coronary artery disease, myocardial infarction; cardiac conditions associated with increased myocardial wall stress, such as arterial hypertension with LV hypertrophy, moderate or severe valvular disease), presence of cardiovascular risk factors (diabetes, hypertension, obesity, smoking, dyslipidemia), significant arrhythmia (e.g. ventricular tachyarrhythmia, AF), renal failure, prior or following chest irradiation, other potentially cardiotoxic drugs (e.g.. trastuzumab, concomitant alkylating or anti-microtubule agents or immunotherapy and targeted therapy), and genetic factors [2, 20]. The onset of cardiotoxicity most frequently occurs within the first year of treatment with the majority of patients remaining asymptomatic; however, overt HF may develop early or late even after decades [21, 22]. Importantly, if HF is detected early, functional recovery, when combined with sufficient HF treatment, is good, whereas late diagnosis is associated with a poorer prognosis [23].

Furthermore, pericarditis, myocarditis and acute arrhythmia as well as ECG(electrocardiogram)-ST changes may also be induced after anthracycline infusion. Right ventricular HF also frequently occurs late after anthracyclines and after combined treatment with radiation [2, 9].

Longer infusion rates of anthracyclines instead of rapid bolus administration can help to avoid cardiotoxicity. Liposomal doxorubicin formulations have been suggested as well as upfront dexrazoxane administration in high-risk patients or with previous high dose exposition [24, 25]. Controversy over dexrazoxane remains due to possible interference with efficacy and high costs. The avoidance of anthracyclines is a current research focus in oncology.

### Recommendations for anthracycline treatment

A baseline echocardiography should be performed to assess LV function or any structural cardiac disease in all patients [6, 20]. After the end of treatment, echocardiography should be repeated. The biomarkers troponin and NT-proBNP (N-terminal pro B type natriuretic peptide) should be tested at baseline and at the end of treatment in all patients [8]. In high-risk cardiac patients, earlier assessment of cardiac function by echocardiography and biomarkers troponin and NT-proBNP should be repeated after every 2–4 cycles, and after end of treatment at 3 months and 6–12 months [6, 8, 20]. Similarly, in higher dose anthracycline regimens, earlier assessment of cardiac function (echocardiography) after a cumulative doxorubicin or equivalent dose of 240 mg/m^2^ should be considered. Troponin measurement has been suggested with each cycle of anthracyclines for high-risk patients and is easily feasible.

## HER2 targeted therapy

Human epidermal growth factor receptors (HERs or ErbBs) have crucial roles in numerous cellular processes. HER2, a ligandless receptor of the HER family, is encoded by the *ErbB2* gene (also known as *HER2/NEU*). Overexpression and/or gene amplification of HER2 is present in up to 20–25% of breast cancers, conferring poor prognosis and diagnosed more often in younger patients or at more advanced stages. Research has led to drugs specifically targeting HER2, such as the monoclonal antibodies trastuzumab and pertuzumab and small molecule tyrosine kinase inhibitors, such as lapatinib, neratinib and tucatinib [26–31]. Further treatment options include the antibody drug conjugates trastuzumab-DM1 and trastuzumab-deruxtecan. Together, these drugs have yielded a massive reduction of recurrence risk in early stage disease and a prolongation of overall survival in patients with metastatic HER2-positive breast cancer. Therefore, cardiac toxicity linked to HER2-directed treatment is of special concern.

Trastuzumab is a humanized monoclonal antibody that targets ErbB2, binding to its extracellular domain IV. It can cause cardiotoxicity that spans from asymptomatic decreases in LVEF to overt congestive HF, especially after previous exposure to anthracyclines or a short time (3 weeks vs. 3 months) between anthracycline and anti-HER2 treatment [32, 33]. The mechanism of anti-HER2 drug-induced cardiotoxicity includes structural and functional changes in contractile proteins and mitochondria, but it rarely leads to myocardial cell death, explaining the potential for reversibility [34]. The inhibition of the neuregulin-1/ErbB2 axis weakens myocardial repair mechanisms making it vulnerable to myocardial injury [35]. The role of neuregulin‑1 in the modulation of doxorubicin-induced oxidative damage with an impact on antioxidant enzymes was demonstrated, suggesting that trastuzumab acts as a modulator of anthracycline-related toxicity in a “dual hit” fashion [36].

Important factors for anti-HER2 drug-induced cardiotoxicity are of high risk in previous cardiovascular disease (very high in pre-existent HF or cardiomyopathy, high after myocardial infarction or in severe valvular disease, reduced LVEF < 50%), and medium risk even in borderline LVEF, arterial hypertension, obesity, smoking, chronic kidney disease, older age > 65 years (high risk > 80 years) or after radiation to the chest [2, 20, 37]. The implementation of different cardiotoxicity surveillance protocols has been proposed [2, 3, 6, 20, 38].

Trastuzumab cardiotoxicity typically already manifests during ongoing treatment but may also present after 1 year. Although trastuzumab-associated cardiotoxicity is not considered to be cumulative dose-related, a doubled rate of LV dysfunction was reported in treatment for 24 months compared to 12 months [39, 40]. In patients with HER2-positive breast cancer receiving adjuvant trastuzumab, cardiotoxicity was the most common reason for treatment interruption in 13.5% of patients (30% for HF and 70% for asymptomatic LVEF decline). In breast cancer patients who developed HF, an association between treatment with ACE inhibitors and beta-blockers and LVEF recovery at 12 months was seen, a further rechallenge with trastuzumab did not necessarily lead to redevelopment of HF, although the risk is in general highly increased [21, 41–43].

Given the major oncological advance that HER2-targeted therapies constitute, the potential negative impact on survival when delaying or discontinuing these therapies should always be considered [44]. The SAFE-HEaRt is the first trial to prospectively examine the safety of HER2-targeted therapies in breast cancer and already compromised cardiac function, a situation often encountered in everyday clinical practice [45, 46]. Importantly, the current results support continuation of HER2-targeted therapies in asymptomatic patients with LVEF 40–49% with cardioprotective HF medication in close cardiology collaboration.

The smaller SCHOLAR study also showed that it is feasible to continue trastuzumab uninterrupted in patients with mild cardiotoxicity (LVEF 40–50% or drop ≥ 15% from baseline), when administered in combination with ACE inhibitors and/or beta-blockers in a cardiology setting performing intensified monitoring, such as a cardio-oncology service [47]. The risk of moderate to severe HF is approximately 10%, and LVEF improved following trastuzumab discontinuation. In these studies, however, 90% of patients were able to complete the trastuzumab therapy and LVEF frequently improved with cardiac medication. As single arm trials, neither SCHOLAR nor SAFE-HEaRT can definitely evaluate if the cancer benefit outweighs the risk of lasting HF when continuing trastuzumab. Therefore, large randomized controlled trials are warranted. Based on the available studies the prescribing information of trastuzumab recommends assessing LVEF before initiation and at regular intervals during treatment. The reversibility of LV dysfunction and the opportunity to resume trastuzumab administration after improvement with HF treatment needs to be evaluated promptly and management should be individualized considering each patient’s characteristics [2, 38, 43, 48]. The cardiotoxicity risk of other anti-HER2-targeted therapies (e.g. lapatinib, pertuzumab, trastuzumab-emtansine, trastuzumab-emtansine (T‑DM1)) appears similar to that of trastuzumab and different combinations are currently applied [2, 37, 49, 50].

### Recommendations for HER2-targeted therapies

Baseline risk factors should be assessed in all patients without delaying the begin of oncological therapy [20]. Pre-existent cardiac diseases or cardiac risk factors such as hypertension, diabetes or older age, borderline LVEF, previous cardiotoxicity with e.g. anthracyclines, chest radiation, pose an increased risk of cardiotoxicity. Serial monitoring by echocardiography including LVEF (3D if available) and global longitudinal strain (if available), as well as biomarkers (NT-proBNP and troponin) and symptom assessment should be implemented [2, 3, 6, 8, 20, 38]. The surveillance frequency depends on the baseline risk factors and any cardiac symptoms [6, 51].

In low-risk patients, echocardiography and biomarkers should be obtained every 3 months (at every 4 cycles of trastuzumab) [2, 6, 38]. In low-risk patients without cardiac history, it has been suggested that cardiac symptoms follow-up with biomarkers alone (NT-proBNP, troponin) may be sufficient, also especially during the corona virus (COVID-19) pandemic [50, 52]. In high-risk patients, frequent echocardiography monitoring and biomarkers are recommended every 2–3 cycles (about 6–9 weeks). In the case of LVEF drop, strain impairment > 15% and/or biomarker increases, prompt initiation of HF medication (ACEI/ARB, betablockers) is recommended [2, 8, 48, 53]. Controversies remain about the efficacy of early preventive treatment at the begin of chemotherapy if there is no cardiac risk [54].

## Hormone modulating endocrine therapies

Breast cancer in women and prostate cancer in men are the most common types of cancer in Europe [55]. Endocrine interventions are cornerstones in their treatment and cardiovascular side effects become more important due to increasing incidence and improved survival [56–59]. Approximately two thirds of breast cancers are estrogen receptor positive.

Tamoxifen, a selective estrogen receptor modulator (SERM), reduces the risk of breast cancer recurrence and improves survival [54]. Although tamoxifen has been associated with a reduction in acute myocardial infarction, infarct-related mortality and lower rates of death from ischemic heart disease [60], extended tamoxifen treatment over 3–4 years has recently been demonstrated to be associated with an increased risk of stroke or TIA (HR 2.00, 95% CI 1.05–3.84), pulmonary embolism and venous thrombosis [56, 60–62]. Furthermore, an increase in body fat, hepatic steatosis, serum lipids/triglycerides and risk of diabetes has been reported with SERMs [56]. Aromatase inhibitors (AIs), such as anastrozole, letrozole and exemestane have further improved the cancer-specific outcome in postmenopausal women [63, 64] and were associated with less thromboembolic events [65]; however, an increased risk for acute ischemic heart disease (HR 2.03; 95% CI: 1.15–3.58) and arrhythmias were reported after long-term AI treatment over more than 4 years [62]. The estrogen receptor downregulator fulvestrant has been associated with less risk of cardiotoxicity, but some possible risk of hypertension [56] and ovarian function suppression, e.g. with triptorelin, has also been associated with risk of hypertension [56].

Prolongation of QTc and possible torsade de pointes ventricular tachycardia present an additional possible side effect of estrogen modulators. Despite the low absolute numbers of QTc prolongation occurrence, this is potentially harmful especially in patients with pre-existing cardiac arrhythmia and possible drug interactions with comedication that might affect the QTc time, e.g. antiarrhythmic agents, or CDK4/6 inhibitors (especially, ribociclib) in metastatic breast cancer [66].

Similarly, androgen blockade in men, especially with LHRH antagonists or abiraterone, is associated with QTc prolongation and has been related to an increased risk for supraventricular arrhythmia and conduction disease [56, 67].

Several other cardiovascular side effects of androgen deprivation therapy, such as hypertension, hypokalemia and edema may appear based on the resulting mineralocorticoid excess. Furthermore, androgen blockage was repeatedly shown to be associated with diabetes and dyslipidemia [68]. Prospective cohort studies and meta-analyses showed conflicting results concerning an increased risk of cardiovascular diseases [69, 70]. In patients without pre-existing cardiovascular disease androgen deprivation was associated with a 27% increased risk for HF, whereas in patients with pre-existing cardiovascular disease androgen blockade was associated with higher rates of arrhythmia and conduction disorders [71]. The LHRH agonists/antagonists are related to weight gain, hypertension, metabolic syndrome and coronary artery disease [56]. In the recent PRONOUNCE study of prostate cancer patients with concomitant atherosclerotic cardiovascular diseases, major adverse cardiovascular events at 1 year occurred in 5.5% of patients assigned to the GnRH antagonist degarelix and similarly in 4.1% assigned to the GnRH agonist leuprolide [72].

### Recommendations for hormone modulating therapy

The cardiac risk profile should be optimized and monitored in all patients receiving a long-term endocrine therapy [56]. Statins, antihypertensive medication and platelet inhibitors should be initiated when indicated in addition to promoting a healthy lifestyle, non-smoking and exercising.

## VEGF and tyrosine kinase inhibitors

Tyrosine kinases (TKs) are often overexpressed or mutated in cancer, a rationale to the development of tyrosine kinase inhibitors (TKIs), which are a class of small molecule drugs [73, 74]. The primary mechanism of action is through competitive inhibition at the adenosine triphosphate (ATP) binding pocket, leading to mitochondrial energy depletion stopping signaling that results in cellular proliferation [74]. While TKIs have been beneficial in improving patient outcomes, clinical trials have documented multiple cardiotoxicities. The phenotype of the cardiotoxicity varies across the available TKIs and the TKs targeted, indicating that no single mechanism is responsible for driving adverse reactions [73–75]. Below we highlight cardiotoxicities often reported with the use of TKIs.

- Hypertension, ischemia, left ventricular dysfunction, HF

One of the most common cardiovascular side effects reported in clinical trials using TKIs is the development of hypertension in up to 80% of patients, with an incidence of symptomatic HF and LVEF decline in 2–15% with sunitinib [2, 38, 73, 74]. The risk of arterial thrombotic events (coronary/myocardial infarction, stroke, peripheral arteries) is increased threefold, especially in pre-existing atherosclerosis [76, 77]. TKIs targeting the vascular endothelial growth factor receptor (VEGFR-TKIs), such as sunitinib, ponatinib, pazopanib and sorafenib are commonly associated with severe hypertension as well as the anti-VEGF monoclonal antibody bevacizumab [73–78]. VEGF plays an essential role in maintaining the blood supply to solid tumors as they grow, so harnessing the ability to cut off angiogenesis and an essential cessation of the tumor blood supply is a strong therapeutic target. Hypertension during anti-VEGFR-TKI therapy is dose-dependent with a rapid onset of just hours or days after the start of treatment and has been related to the release of the vasoconstrictive agonist endothelin [73, 76, 79]. Clinical trials using sunitinib or sorafenib especially in renal cell cancer highlight the importance of monitoring the development of hypertension. Sorafenib inhibits a wide range of TKs including VEGFR‑2, VEGFR‑3, PDGFR (Platelet-derived growth factor receptor), c‑KIT (stem cell factor receptor), RAG (recombination activating gene), and BRAF (B‑Raf proto-oncogene). It is approved for unresectable hepatocellular carcinoma and advanced renal cell carcinoma. In trials, 17% of patients developed hypertension with sorafenib and 3% had an incidence of cardiac ischemia or myocardial infarction [75]. Vandetanib, used in thyroid cancer presents a high rate of hypertension and also QT prolongation, similarly to lenvatinib [80].

Close monitoring of the individual patients is important based on cardiac risk factors, with especially high risk in pre-existing coronary disease, hypertension, or other cardiovascular diseases [20]. Starting adequate antihypertensive treatment to avoid LVD, HF, myocardial ischemia, venous and arterial thromboembolic events, and acute coronary syndromes is crucial. Diltiazem should not be used in patients with TKIs due to possible interaction.

- Arrythmias, AF and QT prolongation

A recent pharmacovigilance study provided evidence that several anticancer drugs represent potential independent risk factors for the development of AF [81]. While both atrial and ventricular arrythmias have been reported with TKI treatment, AF (AF) is more common.

Ibrutinib has revolutionized treatment for several B‑cell malignancies, such as chronic lymphocytic leukemia, mantle cell lymphoma or Waldenstrom’s macroglobulinemia, and inhibits Bruton’s TK protein. Ibrutinib is associated with the development of AF and other types of atrial arrhythmia [82, 83]. A study encompassing 4 large randomized controlled trials reported that at the 36-month follow-up, there was an incidence of 10.4% for AF that was increased with the duration of the treatment [84]. A potential mechanism for AF during ibrutinib is the inhibition of Bruton’s TK protein and tec protein-TK inhibition, which leads to a decrease in phosphoinositide 3‑kinase (PI3k)-Akt activity. Ventricular arrhythmias have also been reported in patients with ibrutinib, e.g. in the HELIOS trial (7 occurrences), as well as other arrhythmias overreported in the international pharmacovigilance database of real-life data [82]. Ibrutinib has also been related to bleeding events, which is important in anticoagulation due to AF [82, 84]. Sorafenib has also been associated with AF (incidence 5.1%), as well as ponitinib [73–75]. QT prolongation has been reported with dasatinib, sunitinib, vandetanib and nilotinib, which now has a black box warning for both QT prolongation and sudden cardiac death [85]. Studies have reported up to 26% of patients having QT prolongation longer than 30 ms with nilotinib treatment.

### Basic science—mechanistic insights

Cardiotoxicities are multifactorial due to multiple targets of TKIs. In mice, sorafenib led to myocyte death due to necrosis [86]. Mice with myocardial ischemia and sorafenib developed pathological hypertrophy and had a significant increase in mortality [86]. Concomitant administration of metoprolol reversed the mortality increase, counteracting sorafenib-induced myocyte death [86]. In a direct assessment on human myocardium, sorafenib treatment affected contractility and decreased the concentration of systolic cytoplasmic calcium [87]. Sunitinib is a TKI targeting platelet-derived growth factor receptors (PDFGR) and VEFGRs on both human myocardium and isolated mouse cardiomyocytes. Sunitinib had a dose-dependent effect leading to worsening contractile function in human myocardium and had a negative impact on calcium homeostasis and sarcomere shortening in isolated mouse cardiomyocytes [88].

### Recommendations for TKIs

Due to exacerbation of pre-existing CVD, direct myocardial toxicity, and hypertension, 5–10% of patients receiving VEGFi (vascular endothelial growth factor inhibitors) TKIs develop LVD [6, 74]. Therefore, a standardized baseline CV risk assessment prior to starting anti-cancer therapy is crucial. Specific evidence-based and practical risk assessment tools have been developed which are easy to use in clinical routine [20]. Echocardiography should be considered early after treatment initiation (2–4 weeks) in high-risk patients, and in all patients every 4 months during the first year. In asymptomatic patients after the first year, echocardiography should be performed 1–2 times/year. Natriuretic peptides (NTproBNP, BNP) should be measured at baseline, followed by at least 3‑monthly measurements depending on CV risk stratification. Markers of cardiac injury, such as troponins are less thoroughly validated but may be considered as well. Daily blood pressure measurements (at home) and monthly ECG recordings should be performed. Initiation of an ACEI/ARB, beta-blocker and statin for cardioprotection prior to starting TKI treatment is a potential option but warrants further investigations. Diltiazem or verapamil are not recommended due to interactions.

Taken together, patients receiving treatment with TKIs should be closely monitored and promptly treated for any acute negative response, such as hypertension or ischemia, and any adverse effects that may develop after a longer duration of treatment, such as AF or HF.

## Immune checkpoint inhibitors

Immune checkpoint inhibitors (ICIs) are antibodies that block negative regulators of the T cell immune response, including cytotoxic T lymphocyte-associated protein‑4 (CTLA-4), programmed cell death protein‑1 (PD-1) and PD‑1 ligand (PD-L1) [89]. ICIs have revolutionized therapies in an increasing number of cancers entities or metastatic settings with an impressive improvement in overall survival with often many years of remission in metastatic melanoma, lung cancer, or refractory Hodgkin’s lymphoma [90–93]. Currently, 7 ICIs are FDA approved (10 in EU, Japan and China) with presently over 50 indications and rapidly expanding, e.g. gastric, colorectal, renal cell, laryngeal, breast cancer and urogenital; however, the augmented immune response has led to a range of immune-related adverse events (irAE), e.g. interstitial pneumonia, colitis, hypothyroidism, hypophysitis, hepatitis, cutaneous dermatitis, nephritis, uveitis, myocarditis, myasthenia gravis and myositis. CTLA‑4 inhibitors include ipilimumab, PD‑1 inhibitors nivolumab or pembrolizumab, and PD-L1 inhibitors avelumab, atezolizumab or durvalumab.

ICI-associated cardiovascular toxicity was rare in trials or pharmacovigilance databases (1% with up to 2.4% in ICI combinations), but potentially life threatening in fulminant autoimmune myocarditis (25–>50% mortality rate), with an increasing incidence of cardiac events reported in growing use of ICIs [94–96]. A higher real-life rate of cardiovascular events was assessed in a recent Danish registry in lung cancer or melanoma patients (1-year absolute risk of cardiac events of 9.7% and 6.6% with PD‑1 inhibitors, respectively, and melanoma 7.5% with CTLA‑4 inhibitors) [97]. Statistically, cardiac side effects occur early, with a median time to onset of 27–65 days (range 2–454 days) [95, 96, 98]. Approximately 2/3 of ICI-associated myocarditis cases were evident after the first or second ICI dose, or within the first 6–8 weeks of therapy [99]. Combination immunotherapy (CTLA‑4 with PD‑1 inhibitor) is a predominant risk factor for ICI-associated myocarditis. The combination of nivolumab and ipilimumab conferred a 4.74-fold risk of developing myocarditis compared with treatment with nivolumab alone, with more severe grade and higher fatality, as well as higher rates of other concomitant immune-induced effects ,such as myasthenia gravis or myositis [100].

The regional distribution of ICI cardiotoxicity and local inflammation may occur in different variations and severity, and include pericarditis and/or pericardial effusion, coronary or other arterial vasculitis, e.g. temporal arteritis, coronary acute plaque rupture and myocardial infarction (myocardial ischemia, subendocardial necrosis), takotsubo syndrome, arterial vasospasm, arrhythmias, high-grade AV block or acute HF [101]. The typical immune-mediated, lymphocytic acute myocarditis may appear as patchy, edematous, and necrotizing. Elevated troponin was observed in 10% of patients with ICI also without symptoms, which might reflect subclinical cardiac dysfunction due to ICI-induced myocardial inflammation [99, 102]. In the presence of other irAEs in a patient with cardiac symptoms, elevated biomarkers, new ECG or echocardiography findings, the probability of ICI-associated myocarditis increases [102, 103]. Importantly, a normal LVEF does not rule out ICI myocarditis, as LVEF was observed as normal in a very high percentage (38%) of cases. Echocardiography may show diffuse LV systolic dysfunction or new regional wall motion abnormalities at development of myocarditis. Preservation of normal cardiac dimensions may be suggestive of an acute process, whereas remodeling and dilatation suggest a chronic myocardial process.

Cardiac magnetic resonance imaging (CMR) is important for tissue characterization and identification of myocarditis; however, not always reliable [104–106]. Indeed, CMR may be unremarkable in a substantial proportion of patients (in up to 50% of cases) and repetitive imaging may increase sensitivity [106]. The presence of LGE increased from 21.6% in CMR performed within 4 days of admission to 72.0% on day 4 or later [106]. Of course, this will cause difficulties in available resources, not only in Austria. In patients where CMR is contraindicated or not available, a cardiac FDG-PET-CT (fluorodeoxyglucose-positron emission tomography-computed tomography) is an alternative image modality to assess inflammation [104]. The gold standard is the histopathological diagnosis by endomyocardial biopsy, and should be pursued in experienced centers, considering the potential consequences of immunosuppression treatment and/or withholding life-saving ICIs [104, 107]. It is essential to clarify the diagnosis of myocarditis, as potentially life-threatening, and the possible rechallenge with ICI after recovery [107]. Furthermore, as was identified in a pharmacovigilance database, patients can suffer from concomitant coronary artery disease, progression of atherosclerosis with plaque rupture induced by ICI inflammation, or vasculitis and ICI-associated myocarditis, so that a biopsy can be performed during angiography in symptomatic patients with troponin, ECG and/or echocardiography abnormalities [104, 108].

ICI myocarditis is usually, but not always, accompanied by elevations in serum biomarkers of myocardial injury (troponin) and LV stress (NT-pro-BNP or BNP) [8, 96, 104]. Biomarkers are not highly specific, at present troponin is considered more specific for myocarditis [8, 99, 104]. Troponin was elevated in 94% of ICI myocarditis [96]. ICI-associated myocarditis can present with various forms of arrhythmia including AF, ventricular arrhythmias and conduction disease, therefore strict ECG monitoring is indicated [98–101, 108, 109].

### Recommendations for surveillance and treatment of ICI myocarditis

Currently, no prospective trial data are available. Recently, surveillance and immunosuppressive treatment algorithms have been suggested [16, 38, 107, 110, 111].

Prior to initiating ICI, baseline cardiac history and cardiovascular risk factors should be assessed [20]. Baseline troponin, NT-pro BNP, ECG and echocardiography prior to ICI initiation should be obtained in all patients for comparison reference in case of cardiac complications. After ICI initiation, troponin measurement has been proposed on a weekly basis for 6 weeks, then every 2 cycles, especially in high-risk cardiac patients, e.g. in ICI combination therapies [108].

In asymptomatic patients with elevated troponin on surveillance, a 12-lead ECG, serum CK(creatinin kinase)/CK-MB(creatine kinase myocardial band) and serial troponin should be assessed. Patients should be referred to a cardiologist, and echocardiography or CMR considered. In asymptomatic patients and isolated troponinemia, resuming ICI therapy can be considered if troponin returns to normal within 2 weeks and no cardiac pathologies are present in work-up [16, 102].

In symptomatic patients (e.g. chest pain, dyspnea, palpitations, presyncope, syncope), abnormal ECG or echocardiography or CMR findings, hold ICI treatment and admit patient to hospital with cardiac monitoring. Myocardial infarction and other etiologies should be ruled out (coronary CT and/or angiography), echocardiography and CMR obtained, and endomyocardial biopsy considered (even with unremarkable echocardiography or CMR if clinical suspicion is high) [110].

In hemodynamically stable ICI myocarditis without arrhythmia and concomitant signs of potential fulminant course (myositis, myasthenia) start patients with i.v. methylprednisolone 500–1000 mg i.v. bolus for 3 days and continue with 2 mg/kg/day [16, 38, 100, 110, 111]. If abnormalities resolve, methylprednisolone should be tapered over at least 4 weeks. If no improvement in 24 h or patient unstable (hypotension, arrhythmia, or sudden EF decrease), a transfer to ICU and begin i.v. methylprednisolone at 1 g daily is recommended. If the patient continues to be unstable, adding alternative agents, such as antithymocyte globulin (ATG), tacrolimus, infliximab, mycophenolate mofetil, or rituximab should be considered early. Recently, especially abatacept has been reported for severe immune myocarditis, also in an experimental concept, and tocilizumab [111–114]. Pacemaker insertion may be necessary due to high degree AV block. Importantly, treatment should not be delayed and contact to experienced centers sought early.

## New immune therapies, chimeric antigen receptor (CAR) T-cell therapies

Immunotherapy is revolutionizing oncology by harnessing the native immune system for treating even advanced malignancies. The emerging chimeric antigen receptor T cell (CAR‑T, e.g. tisagenlecleucel, axicabtagene ciloleucel) therapies induce tumor cell apoptosis, but may provoke severe cytokine release syndromes (CRS) with acute HF, hypotension, multiorgan dysfunction and high fatality rates [115–117]. Interleukin‑6 (IL-6) is a primary driver of inflammation in CRS, and also associated with cardiovascular complications [116].

Bispecific T cell engager therapy (BiTE, e.g. blinatumomab) showed grade 3–4 CRS in 19% of patients [116]. Surveillance for adverse cardiovascular effects is warranted in these evolving immunotherapies, and further knowledge is needed.

## Alkylating, antimicrotubule agents, fluoropyrimidines

Other common chemotherapies bearing a cardiotoxicity risk are the alkylating substances cyclophosphamide, ifosfamide, the platinum-based cisplatin and carboplatin, the antimicrotubule taxanes, i.e. paclitaxel and docetaxel, and the antimetabolites fluoropyrimidines (5-FU fluorouracil) ([118]; Table 1).

Cardiotoxicity of cyclophosphamides is mainly associated with higher doses, previous anthracyclines, radiation, trastuzumab combinations and older age, with an incidence of HF up to 28% [118]. Cisplatin or ifosfamide can induce myocardial ischemia and coronary plaque rupture. Combining docetaxel and cyclophosphamide is in general less cardiotoxic than a combination with anthracyclines. The non-anthracycline regimen in breast cancer with docetaxel, carboplatin, and trastuzumab (TCH) was less cardiotoxic than with anthracycline [119].

Cancer including radiation and chemotherapies may lead to accelerated coronary atherosclerosis and a prothrombotic state [5]. The anti-metabolites 5‑FU and the oral prodrug capecitabine can frequently cause chest pain usually early within hours or days after initiation of 5‑FU, and a work-up for ischemia should be initiated. ECG changes and/or biomarker increases were found in up to 43% of patients, the risk of mortality ranged from 2–13% [118]. Risk factors include high dose of 5‑FU or continuous infusion, pre-existing cardiovascular disease and concurrent other chemotherapies. The capecitabine-induced cardiotoxicity was lower and ranged between 3–9%. Coronary vasospasm is also caused via endothelial injury and vasoconstriction by 5‑FU infusion. Second to anthracyclines, 5‑FU has been considered as one of the most common cardiotoxic agents [120, 121].

In taxanes, myocardial ischemia and infarction have been reported less frequently in 3% of patients receiving paclitaxel, and after docetaxel in 1.7%, as well as HF in 2.3–8% [118].

### Recommendations during cisplatin or fluoropyrimidine treatment

In chest pain, new ECG changes, elevated biomarkers with troponin, a coronary syndrome needs to be excluded. Higher risk is present in patients with previous cardiovascular disease and atherosclerosis risk factors.

## Proteasome inhibitors (PI) and immunomodulatory agents (IMID)

Inhibition of proteasome blocks cell proliferation and induces apoptosis in tumor cells [118]. Bortezomib, carfilzomib and ixazomib are targeted therapies for multiple myeloma. Proteasome inhibition perturbs protein homeostasis and function of cardiomyocytes, significant in already present cardiac stressors. Especially carfilzomib, an irreversible PI, has a higher risk of cardiovascular toxicity including myocardial ischemia or infarction, LV dysfunction and symptomatic HF (about 7%) [122]. Cardiotoxicity occurred early during PI treatment and was largely reversible with (intermittent) cessation and initiation of HF medication [123]. A high risk is present in previous CVD, older age, reduced or borderline LVEF, and previous venous thrombosis or embolism [20]. Elevated BNP levels predict major adverse cardiac events (MACE) [8, 124].

The immunomodulators lenalidomide, as well as thalidomide or pomalidomide, are associated with an increased risk of venous thromboembolism, acute coronary artery thrombosis, as well as AF or bradycardia [125–127]. Combination regimes with bortezomib and lenalidomide are a standard therapy for multiple myeloma, or with carfilzomib and lenalidomide in relapsed or refractory myeloma. Main cardiovascular complications of the combinations of PI and IMIDs are LVD, HF, ischemia, infarction, atrial and ventricular arrhythmia, venous thromboembolism and arterial thrombosis [2, 81, 123].

### Recommendations for PI and IMID

Due to relatively high cardiovascular event rates, a baseline echocardiography is recommended in all multiple myeloma patients scheduled to receive a PI, also assessing for cardiac AL amyloidosis, and surveillance in medium to high-risk patients especially in carfilzomib [6, 20]. Echocardiography is strongly recommended if new cardiac symptoms appear. Measurement of BNP at baseline and during the first few cycles in patients receiving PIs should be considered [8]. Thromboprophylaxis is recommended in lenalidomide-based regimens.

## Cyclin-dependent kinase 4/6 inhibitors

CDK 4/6 inhibitors are a major milestone for hormone receptor-positive and HER2-negative metastatic breast cancer [128]. Palbociclib, ribociclib, and abemaciclib, have significantly improved progression-free survival combined with endocrine therapy [125]. CDK 4/6 inhibitors, especially ribociclib, have been associated with QT prolongation [129].

### Recommendations for CDK 4/6 inhibitors

For ribociclib, ECG monitoring is recommended (baseline, day 15, and begin of cycle 2) with QTc value of ≤ 450 ms at initiation. Other QT prolonging concomitant drugs should be considered in CDK 4/6 inhibitors, as well as electrolyte imbalance, e.g. in diarrhea or emesis.

## Therapy of cardiotoxicity and general recommendations in oncological agents

In all cancer patients treated with potentially cardiotoxic therapy, it is generally recommended to strictly control cardiovascular risk factors without delaying the oncological therapy [2, 20, 38].

In potential CTRCD and HF risk, LVEF should be determined before and periodically during treatment, usually by echocardiography and biomarker surveillance ([6, 8, 38, 51]; Table 2). If LVEF deteriorates, LVEF should be reassessed after 2 weeks before stopping the life-saving oncological treatment, especially in asymptomatic patients [2, 6]. If LVEF deteriorates > 10% to values < 50%, or in impaired strain (> 15% change in GLS), ACE inhibitors or ARB and beta-blockers are promptly recommended to prevent further deterioration as these patients are at high risk to develop manifest HF [21, 23, 48, 130–132]. Patients with asymptomatic or symptomatic deterioration of LVF during or after tumor treatment should be treated according to current HF guidelines [130]. Optimal and early initiation of HF treatment is associated with improved LV function after chemotherapy [21], Currently, sacubitril/valsartan in cardiotoxicity has only been evaluated in few studies [133, 134].

**Table 2:** Summary of guidance for echocardiography timelines, including LVEF and GLS where feasible (modified from [6, 51, 135]). This is an open access article under the CC BY license (http://creativecommons.org/licenses/by/4.0/). This Table is not included under the Creative Commons CC BY license of this publication

	Baseline	During therapy		After completion	
			Comment		Comment
Trastuzumab (in early invasive disease)	Yes	Every 4 cycles	Every 2 cycles in high risk, every 3 cycles in medium risk	6 months after final cycle	3 and 12 months after final cycle in high risk
Trastuzumab in metastatic disease (long-term therapy)	Yes	Every 4 cycles	Every 6 months when stable	Not indicated unless symptomatic	–
			More frequent in medium to high risk: every 2–3 cycles		
Anthracyclines	Yes	After completing cumulative dose of 240 mg/m^2^ doxorubicin	Every 2 cycles in medium to high risk	6–12 months after final cycle (depending on risk)	Reassess after 5 years (earlier in high risk)
VEGF and Bcr-Abl TKIs	In high-risk patients	Every 4 months during the first year	Every 6–12 months, when long-term therapy is necessary	No clear recommendations	–
Proteasome inhibitors	Yes	Every 6 months	Look for signs of amyloidosis	No clear recommendations	–
Immune checkpoint inhibitors	Yes (depending on baseline risk)	Immediately when cardiac symptoms occur. Every 6–12 months in long-term in high risk	CMR if myocarditis suspected, consider EMB	No clear recommendations	Consider in high risk

*GLS* global longitudinal strain, *LVEF* left ventricular ejection fraction, *VEGF* vascular endothelial growth factor, *TKI* tyrosine kinase inhibitor, *CMR* cardiac magnetic resonance imaging, *EMB* endomyocardial biopsy

In general, patients with cancer therapy cardiotoxicity should be referred to a cardio-oncology specialist in close cooperation with the oncology team to commonly assess the duration or even interruption of chemotherapy until the patient is clinically stable. It is important to critically discuss risk versus benefits of further treatment with the previous cancer regimen based on clinical factors, such as LV dysfunction, NYHA status, prognosis of the disease, and alternative treatment options considering the spectrum of side effects.

In case an anticancer drug previously causing cardiotoxicity is readministered, it is recommended that concomitant cardioprotective therapy with ACEI or ARB and beta-blockers is provided and continued indeterminately.

## Radiation therapy: early and late cardiotoxicity

Irradiation of the heart is unavoidable when the target volume is close, as in left breast cancer or frequently mediastinal in e.g. Hodgkin’s lymphoma or thymus cancer. After irradiation, patients are at risk of long-term radiation-induced heart disease (RIHD, e.g. valvular, pericardial, myocardial, coronary disease, conduction system) and peripheral artery disease depending on the field of radiation [136, 137]. Risk factors for RIHD include anterior or left chest radiation, a high cumulative radiation dose, age < 50 years, tumor in or near to the heart, lack of shielding, concomitant chemotherapy particularly with anthracyclines, smoking and medical comorbidities (diabetes mellitus, hyperlipidemia, hypertension, and obesity) [137]. The effects of radiation of the heart typically manifest years after exposure [136–138]. Current strategies to prevent cardiac impairment are limiting cardiac exposure by shielding and modern radiation techniques, e.g. breathing maneuvers.

LVD and HF are relatively common serious side effects of radiation therapy. The actual incidence of radiation-induced cardiotoxicity is difficult to evaluate due to long delay between exposure and clinical manifestation of heart disease, concomitant cardiotoxic chemotherapy and continuous improvements in radiation techniques. Studies found a relative risk of fatal cardiovascular events of 2.2–12.7 in survivors of Hodgkin lymphoma and 1–2.2 in patients with breast cancer [136, 139, 140]. In breast cancer treated in the era 1980–2000, the risk of cardiotoxicity was highest in patients treated with both left breast radiotherapy and cardiotoxic chemotherapy, suggesting a synergistic effect on cardiac risk. LVD is generally observed when radiotherapy is combined with anthracyclines.

In the acute phase, acute myocarditis related to radiation-induced inflammation with transient repolarization abnormalities and mild myocardial dysfunction can occur. In the long term, diffuse myocardial fibrosis with relevant systolic and diastolic dysfunction (including restrictive cardiomyopathy) and conduction disturbances can occur, e.g. AV block necessitating pacemaker [136–138].

Supradiaphragmal and, in certain patient groups, even infradiaphragmal radiotherapy may be associated with a higher incidence of ischemic heart disease through the development of severe atherosclerotic and non-atherosclerotic disease. Radiation-related cardiac disease in patients with lymphoma typically manifests 15–20 years after the initial treatment, and younger patients are more susceptible than older patients [139]. Survivors of Hodgkin’s lymphoma have a 4–7-fold increased risk of CAD compared with the general population and a cumulative incidence of CVD up to 50% 40 years after treatment. Presentation of CAD is more often atypical with diffuse sclerosis [137, 138]. The prevalence of silent ischemia may be higher than in conventional CAD, possibly because of concomitant neurotoxicity of radiotherapy or chemotherapy affecting the patient’s perception of angina. Sudden cardiac death in irradiated patients has been reported and linked to diffuse intimal hyperplasia of all coronary arteries or to significant left main coronary artery stenosis [136–138]. Ostial lesions are frequent and a potentially life-threatening complication. The most exposed coronaries are the left anterior descending artery during left breast irradiation and the left main stem, circumflex and right coronary arteries during treatment for Hodgkinʼs lymphoma. A higher prevalence of stress test abnormalities and impaired LV strain has been found among women irradiated for left breast cancer compared with right-sided cancer [141].

Radiation-induced valvular heart disease is common, affecting 10% of treated patients, and includes fibrosis and calcification of the aortic root, aortic valve cusps, mitral valve annulus and typically the base and middle portions of the anterior mitral valve leaflets, sparing the mitral valve tips and commissures, allowing distinction from rheumatic disease [137]. Many patients will later need valvular or coronary interventions [142, 143]. Transcatheter aortic valve replacement may be preferable to surgical procedures. In contrast, in mediastinal involvement treated today with 20 or 30 Gy, the 30-year risk may be less increased only by 1.4% [143, 144]. Radiation calcified valve disease and heart failure are frequently encountered years after radiation [145].

Any type of supraventricular arrhythmia may arise acutely, of which AF is the most common. Ventricular arrhythmias can be related to QT prolongation, to acute and chronic toxicity of radiotherapy (mainly LV dysfunction and ischemia) and to predisposing factors. Sinus node dysfunction and conduction defects may arise following radiotherapy and are often permanent [137, 138].

Acute pericarditis with typical chest pain, fever, ST‑T changes and large effusions may develop 2–145 months after thoracic radiotherapy, with an absolute cumulative incidence of 2–5%. Treatment of pericardial effusion consists primarily of non-steroidal anti-inflammatory drugs and colchicine. Pericardiocentesis may be required in hemodynamic compromise, followed by surgical pericardial windowing if needed. Chronic thickening and constrictive pericarditis may develop after high-dose radiotherapy [136, 144].

Endothelial damage and thrombus formation may occur also after irradiation of small cerebral vessels, leading to increased carotid stiffness and intima media thickness and direct irradiation to advanced carotid atherosclerosis at around 10 years after radiotherapy. The risk of stroke is increased, at least doubled, after mediastinal, cervical or cranial radiotherapy [146]. Similar consequences are reported for the aorta (porcelain aorta) and other peripheral arteries, including the subclavian and iliofemoral vessels [136, 137, 146]. Also, the mammary artery may be affected, which is relevant in planning of coronary artery bypass surgery [142]. Additional pericardial adhesions and pulmonary irradiation-induced fibrosis lead to higher perioperative complications and bleeding in heart surgery [137, 138, 142]. Newer radiation procedures today lead to significantly lower cardiac side effects.

### Recommendations after radiation therapy

Especially younger survivors of breast cancer, lymphoma or other mediastinal cancers after chest radiation need to be followed for cardiovascular late effects including coronary disease, valve disease and pericardial constriction by echocardiography, clinical symptoms evaluation, stress test and biomarkers after 3–5 years and long term after that, with coronary evaluation by CT or angiography as needed [137, 138]. Measures for cardiovascular disease prevention should be considered early. Risk factors such as high lipids should be treated early by statins, smoking cessation, antihypertensive medication and platelet inhibitors started where indicated. Transcatheter procedures may be preferable to surgical interventions after chest irradiation.

## Survivors of childhood cancer

Survival of childhood malignancies has considerably increased in the last decades with current 5‑year survival rates > 80% [147]. After exposure to cardiotoxic medication and/or radiation early in their lifetime, survivors are at risk for late adverse events. Although many patients after childhood cancer may still die due to recurrence of the primary malignancy or secondary neoplasm, cardiovascular diseases are the most common non-neoplastic cause of premature mortality in this population [148, 149]. Survivors of pediatric cancers have a higher prevalence of cardiovascular risk factors, predominantly hypertension and dyslipidemia, as well as obesity and diabetes, also due to growth factor and other metabolic impairments after chemotherapy or whole body irradiation in hematopoietic stem cell transplantation [150, 151]. Moreover, a 2–5-fold increase of relative risk for cardiovascular diseases (7–10% cumulative long-term risk) has been reported in these patients [149, 151, 152]. Cardiomyopathies and venous thromboembolism account for more than 50%, with highest risk after exposure to anthracyclines, chest or cerebral radiation [151]. Although Scandinavian data have demonstrated a reduction of the increased cardiovascular risk over time, others have shown that the risk remains beyond the age of 60 years and an increase with age [149, 151, 152]. Based on these observations, it is recommended that adult survivors of childhood cancers should be regularly screened for traditional cardiovascular risk factors and examined for cardiomyopathy and coronary disease [2, 6, 147, 153, 154].

Surveillance by NT-proBNP may have only moderate sensitivity and troponin less useful, therefore regular echocardiography at intervals of no more than 5 years is advised (Table 2; [2, 8, 147, 153, 155, 156]). Global longitudinal strain was impaired in 9–30% of childhood cancer survivors even in normal LVEF [157]. Despite lack of randomized trials, asymptomatic reduction of LVEF should be treated with RAAS inhibition and beta-blockers and followed closely [2, 158]. Apart from medical therapy, regular exercise is recommended for survivors treated with anthracyclines and/or chest radiation [153]. Female adult survivors treated with cardiotoxic chemotherapy or chest radiation are strongly advised to consult a cardiologist before planning pregnancy and echocardiography and biomarkers should be considered [6, 8, 153].

### Recommendations in survivors of childhood cancer

Regular follow-up in adult survivors of childhood cancer is advised with ECG, echocardiography and cardiac risk factor treatment, e.g. statins in hyperlipidemia, metabolic syndrome, every 5 years, biomarkers if available and evaluation for early coronary disease at least by the age of 40 years.

## Special aspects

### Long-term cardiovascular monitoring

Survival, especially in breast cancer and lymphomas, has substantially increased over the past decade [159]. It is imperative to raise awareness of possible cardiac sequences among cancer survivors in general, as well as to provide appropriate follow-up in clinical practice, as in survivorship programs managed by oncology or hematology centers, but not always available [2–4]. Cardio-oncology centers should work with such survivorship programs to assure optimal cardiac follow-up after potentially cardiotoxic therapies. Patients should be informed of their increased risk of CVD at the end of their oncological therapy and should be advised and supported to make appropriate lifestyle modifications. They should be instructed to promptly report early signs and symptoms of CVD. A yearly history and physical examination to assess for early signs of CVD, as well as regular follow-up with imaging is recommended in the recent consensus documents [2, 3]. Personalized echocardiographic surveillance and long-term follow-up according to the baseline cardiotoxicity risk (low, medium or high) aligned to therapy-related and patient-related factors is recommended [6, 20].

In breast cancer or Hodgkin’s lymphoma, echocardiography is recommended at 12 months and 5 years post-completion of anthracycline chemotherapy in patients with low or medium baseline risk, in patients with high baseline risk at 6 and 12 months, annually for 2 or 3 years thereafter, and then in 3–5-year intervals for life. In breast cancer asymptomatic patients after HER2-targeted treatment with medium or high baseline cardiotoxicity risk, a follow-up echocardiogram and clinical assessment should be considered 3–6 months and 12 months after the final dose [3, 6, 20, 51]. In patients who require long-term trastuzumab treatment in the setting of metastatic disease, regular long-term echocardiographic surveillance is recommended [3, 6]. Cardiac biomarkers and imaging are synergistic and should be applied together [8]. Biomarker surveillance after anthracycline therapy with BNP/NT-proBNP and troponin is recommended 12 months after the final cycle, in high-risk patients also 3 and/or 6 months after the final cycle. A similar biomarker surveillance after HER2-targeted therapies is recommended [8].

Following radiation, evaluation for CAD and ischemia is recommended in patients with a history of mediastinal radiation as in breast cancer or Hodgkinʼs lymphoma, even if asymptomatic, starting 3–5 years post-treatment and then at least every 5 years thereafter [2, 137, 154]. Patients irradiated for head and neck cancer or lymphoma should undergo cerebrovascular ultrasound screening, especially beyond 5 years after irradiation. Duplex imaging may be considered at least every 5 years or earlier and/or more frequently if the results of the first examination are abnormal. Stringent risk factor management is required to halt plaque progression. Antiplatelet drugs and statins should be considered where indicated.

### Exercise therapy for oncological patients

The efficacy of exercise therapy during and after cancer therapy has been confirmed in a number of trials [160, 161]. Despite some limitations, even prediagnosis exercise exposure is associated with a significant reduction in subsequent cardiovascular events in long-term survivors of primary breast cancer [162]. An analysis of 56 trials involving 4826 participants demonstrated an improvement in quality of life and physical ability during and after an exercise-training program [160]. In particular, aerobic exercise is considered a promising non-pharmacological strategy to prevent and/or treat chemotherapy-induced cardiotoxicity [163]. The importance of exercise as a safe and effective strategy to improve physical, muscular, and cardiovascular fitness in adult patients with cancer improving their quality of life (QOL) has been emphasized [164]; however, the timing, type, intensity, and frequency of exercise that gives maximum cognitive and physical benefit are still unclear. The recent AHA statement introduces the concept of cardio-oncology rehabilitation and points out that low cardiorespiratory fitness is associated with a higher incidence of short-term and long-term treatment-related toxicities (e.g. cardiovascular disease), higher symptom burden (e.g. fatigue) and increased risk of all-cause and cancer-specific mortality in patients with cancer [165]. Ongoing trials will determine the role of exercise to prevent and ameliorate cardiac and other side effects of chemotherapy, e.g. in breast cancer or other cancer entities [166].

## Conclusions and gaps of evidence

As oncology therapies rapidly evolve and cancer survival improves dramatically, cardiac side effects emerge as important determinants of long-term QOL and outcome.

Open questions remain concerning the correct timing and which medication should be administered to avoid or treat overt cardiotoxicity in oncology treatment.

Biomarker elevations need to be differentiated to other cardiac comorbidities (e.g. pulmonary embolism, coronary syndromes, arrhythmias) or volume changes in intravenous infusions, diarrhea or emesis in cancer therapies. Suggested timepoints of biomarker assessment during cancer therapy are summarized (Table 3), although further studies will be needed [8].

**Table 3:** Summary for the guidance of cardiac biomarker measurement timepoints, depending on cardiovascular risk (modified from [8]). This is an open access article under the CC BY license (http://creativecommons.org/licenses/by/4.0/). This Table is not included under the Creative Commons CC BY license of this publication

	Baseline	During therapy		After completion	
			Comment		Comment
*Trastuzumab (in early invasive disease)* (BNP/NT-proBNP, cTn)	Yes	Every 4 cycles (low risk)	Before alternate cycles for 3–6 months and then every 3 cycles for the remaining year 1 in medium risk	Optional 6–12 months after final cycle in low risk	3–6 months after final cycle in medium risk, optional at 12 months
			Before and after every cycle for 3–6 months and then every 3 cycles for the remaining year 1 in high risk		3 and 12 months after final cycle in high risk
*Trastuzumab in metastatic disease (long-term therapy)* (BNP/NT-proBNP, cTn)	Yes	in medium or high risk	Every 4 months in medium risk	Not indicated unless symptomatic	–
			Before every cycle for 3–6 months and then every 3 cycles for the remaining year 1 in high risk		
*Anthracyclines* (BNP/NT-proBNP, cTn)	Yes	Before 5th cycle in low (optional) and medium risk	Before every cycle in medium risk (optional)	12 months after final cycle (low and medium risk)	3 and/or 6, and 12 months after final cycle (high risk)
			Before cycles 2, 4 and 6 in high risk (optional before every cycle)		
*Anti-VEGF therapy* (BNP or NT-proBNP)	Yes	Every 3 months (low risk)	2–4 weeks after starting treatment in medium and high risk	No clear recommendations	–
*Proteasome inhibitors* (BNP/NT-proBNP)	Yes	Consider during first cycles	No clear recommendations	No clear recommendations	–
*Immune checkpoint inhibitors* (BNP/NT-proBNP, cTn)	Yes	Immediately when cardiac symptoms occur	Before doses 2, 3, and 4 in high risk (combination ICI treatment). If normal at dose 4 reduce to alternate doses for 6–12; If still normal, reduce to every 3 doses until completion of course	No clear recommendations	–

*BNP/NT-proBNP* N‑terminal B type natriuretic peptide, *VEGF* vascular endothelial growth factor, *cTn* cardiac troponin

Availability and increasing costs of repeated frequent cardiac imaging, biomarkers and other examinations for cardiovascular surveillance during and after cancer treatments are limiting factors, not only in Austria.

Considering that outcome in cardiac diseases has also strikingly ameliorated in the last decade, patients with pre-existing HF and other CVD are increasingly in need of special cardio-oncology care when developing additional cancer [167, 168]. Baseline assessment of risk factors for cardiotoxicity and regular surveillance during oncological treatment are recommended. Cooperation of oncologists and cardiologists is mandatory to provide optimal care to patients with cancer and/or heart diseases and their follow-up in long-term survivors.

### Outlook in Austria

The necessity of frequent cardio-oncology surveillance with cardiac imaging, biomarkers, ECG and clinical examinations of patients with increasing age and comorbidities opens issues of costs and feasibility with current resources in Austria.

Increased imaging availability of echocardiography, CMR and CCT will be crucial in the future. Reimbursement issues concerning echocardiography and biomarker (troponin, BNP) assessment need to be clarified in the Austrian healthcare system, especially outside of hospitals. Currently, very few specialized cardio-oncology units are available in Austrian hospitals, and further infrastructure and collaboration need to be established [169].

## Abbreviations

ACEI: Angiotensin-converting enzyme inhibitor
AF: Atrial fibrillation
AI: Aromatase inhibitor
ARB: Angiotensin receptor blocker
CAD: Coronary artery disease
CMRI: Cardiac magnetic resonance imaging
CTRCD: Cancer therapeutics-related cardiac dysfunction
CVD: Cardiovascular disease
EACVI: European Association of Cardiovascular Imaging
ESC: European Society of Cardiology
HER: Human epidermal growth factor receptor
HF: Heart failure
HFpEF: Heart failure with preserved ejection fraction
ICI: Immune checkpoint inhibitor
LV: Left ventricle
LVD: Left ventricular dysfunction
LVEF: Left ventricular ejection fraction
MACE: Major cardiac events
RIHD: Radiation-induced heart disease
TKI: Tyrosine kinase inhibitor
